# Pan-cancer and single-cell analysis reveals *FAM83D* expression as a cancer prognostic biomarker

**DOI:** 10.3389/fgene.2022.1009325

**Published:** 2022-12-09

**Authors:** Haiyang Yu, Qinhao Chen, Ziming Wang, Xiaojun Qian, Yueyin Pan

**Affiliations:** ^1^ Provincial Hospital Affiliated to Anhui Medical University, Hefei, Anhui, China; ^2^ The First Affiliated Hospital of University of Science and Technology of China West District, Hefei, Anhui, China; ^3^ Wannan Medical College, Wuhu, Anhui, China; ^4^ Department of Medical Oncology, The First Affiliated Hospital of USTC, Division of Life Sciences and Medicine, University of Science and Technology of China, Hefei, Anhui, China

**Keywords:** pan-cancer, breast cancer, prognosis, FAM83D, plk1, biomarker

## Abstract

**Background:** The family with sequence similarity 83 member D (*FAM83D*) protein is known to play a significant role in many human diseases. However, its role in cancer remains ambiguous. This study aimed to investigate the function of *FAM83D* in a pan**-**cancer analysis, with a special focus on breast cancer.

**Methods:** Samples were collected from The Cancer Genome Atlas (TCGA) and used for bioinformatic analysis. Datasets from the Gene Expression Omnibus (GEO) and Genotype-Tissue Expression (GTEx) databases were also analyzed for verification. The potential value of *FAM83D* as a prognostic and diagnostic biomarker was visualized through R software. The “survival” and “GSVA” package were used for univariate, multivariate and pathway enrichment analyseis. We further analyzed the CancerSEA databases and TISIDB websites for single-cell and immune-related profiling. Lastly, we validated those data *in vitro* using quantitative reverse transcriptase-polymerase chain reaction (RT‒qPCR), cell counting kit-8 (CCK-8), transwell, flow cytometry, and tumorigenicity assays in a murine cell line model.

**Results:** The expression of *FAM83D* in tumor samples was significantly higher than in normal tissues for most cancer types in the datasets. We confirmed this finding using RT‒qPCR in a breast cancer cell line. Analysis of multiple datasets suggests that overall survival (OS) was extremely poor for breast cancer patients with high *FAM83D* expression. The CCK-8 assay demonstrated that MCF-7 cell proliferation was inhibited after genetic silencing of *FAM83D*. Transwell assay showed that knockdown of *FAM83D* significantly inhibited the invasion and migration ability of MCF-7 cells compared to the control. The results of flow cytometry showed that silencing *FAM83D* could block the G1 phase of MCF-7 cells compared with negative groups. The tumorigenicity assay in nude mice indicated that the tumorigenic ability to silence *FAM83D* decreased compared.

**Conclusion:** Results suggest that *FAM83D* expression can serve as a valuable biomarker and core gene across cancer types. Furthermore, *FAM83D* expression is significantly associated with MCF-7 cell proliferation and thus may be a prospective prognostic biomarker especially for breast cancer.

## Introduction

Nearly 10 million people die annually from malignant tumors (Sung et al., 2021). In recent years, the life expectancy of cancer patients has increased in part due to ongoing improvements in treatment strategies and rapid progress in the science and technology sectors. Both the behavioral traits and treatment options of cancer are complex and variable. Certain tumors with various molecular traits and subtypes can result in a poor prognosis for some individuals, especially in breast cancer (BRCA) (Hanahan and Weinberg., 2011; Siegel et al., 2022).

The family with sequence similarity 83 (FAM83) protein belongs to a protein family where in studies have found that many members of the family have carcinogenic effects. For example, FAM83A has been identified as exhibiting a tumor-promoting role in lung cancer and BRCA and can cause epidermal growth factor receptor-tyrosine kinase inhibitor (EGFR-TKI) resistance by activating the phosphatidylinositol-3-kinase-protein kinase B (PI3K-AKT) and mitogen-activated protein kinase (MAPK) signaling pathways (Li et al., 2005; Wang et al., 2015). However, the role of FAM83D, one member of the FAM83 family, in cancer is still unclear. Previous research has revealed that FAM83D is a transactivating factor expressed in the late G1/S phase of the cell cycle and is related to cell growth and the transition from G1 to S phase (Cipriano et al., 2012; Shi et al., 2016). By adversely regulating eurogenic locus notch homolog protein 1 (Notch-1), Mu et al. demonstrated that FAM83D can enhance the invasion and metastasis of colorectal cancer cells (Mu et al., 2017). In addition, suppression of *FAM83D* has been shown to inhibit glioma cell proliferation, invasion, and migration by regulating the Akt- mammalian target of rapamycin (Akt-mTOR) signaling pathway (Li et al., 2022).

Despite these investigations, the pan-cancer function and prognostic value of FAM83D, especially in BRCA, remains unknown. Although some patients with BRCA achieve successful remission and increased survival from chemotherapy, targeted therapy, and immunotherapy regimens, there are still a significant number of patients with BRCA who are subject to poor outcomes, necessitating the urgent development of new, practical, and straightforward therapies. Historically, there have been some indicators for predicting prognosis in BRCA mutation carriers (Lu et al., 2020; Saikia et al., 2021; Zeng et al., 2019). However, several of these biomarkers have low sensitivity or specificity; hence, it is necessary to investigate more accurate biomarkers for predicting the prognosis of patients with BRCA. In this study, using sequencing data from The Cancer Genome Atlas (TCGA) and Gene Expression Omnibus (GEO), we investigated the function and prognostic utility of *FAM83D* expression in breast cancer.

## Materials and methods

### Expression levels and diagnostic value

RNAseq data from TCGA (11,093), GSE93601 (1,110), and GSE70947 (296) datasets in the GEO database were Toil (Vivian et al., 2017) processed and converted to log2 format for analysis. In addition, 179 samples of normal breast tissue were obtained from the Genotype-Tissue Expression (GTEx) database. The Mann‒Whitney *U* test was used to analyze the difference in *FAM83D* expression levels between tumor and normal tissues. R software (× 64, 3.6.3) was used for statistical analysis and visualization. Receiver operating characteristic analysis was performed using the R software “pROC” package to calculate the diagnostic efficacy of *FAM83D* expression. Significance was set at *p* < 0.05.

### Prognosis analysis

The prognostic data for TCGA came from the research archives of Liu et al. (Liu et al., 2018). The data filtering criteria used were as follows: the controlled/normal sample was removed, and the sample with clinical information was retained. Kaplan‒Meier survival analysis was applied using the R “survival” package. Subsequently, *FAM83D* expression data and prognostic survival information from GSE9195 and GSE12276 were validated as independent cohorts. The R “survival” package was also used to perform univariate and multivariate analyses.

### Immunocyte infiltration analysis

The single sample gene set enrichment analysis (ssGSEA) algorithm built into the R “GSVA” package (Hanzelmann et al., 2013) was used to perform immunoinfiltration analysis on 33 cancers in the TCGA dataset. This algorithm uses the specific markers of each type of immune cell as a gene set to calculate the enrichment score of each type of immune cell in each sample and thus infers the infiltration of immune cells in each sample (Bindea et al., 2013). Subsequently, the TISIDB online website (http://cis.hku.hk/TISIDB/) was used to map the relationship between *FAM83D* expression and Immunoinhibitor, Immunostimulator, and major histocompatibility complex (MHC) molecules in different cancer types.

### Differential gene expression and pathway enrichment analysis in BRCA

Single-cell sequencing data from 14 cancers in the CancerSEA dataset were used to assess the association of *FAM83D* expression with 14 cancer functional states. BRCA single-cell sequence data from the GSE77308 dataset were also used. In addition, the R “DESeq2” package (Love et al., 2014) was used for genetic analysis of the differences between high and low expression groups of FAM83D (LogFC = 2, *p* < 0.01) in BRCA. The R “clusterProfiler” (Yu et al., 2012) and “org.Hs.eg.db” packages were used for gene ontology (GO) analysis, Kyoto Encyclopedia of Genes and Genomes (KEGG) analysis, and gene set enrichment analysis (GSEA) pathway enrichment analysis (Walter et al., 2015). The Spearman algorithm built into the R “ggplot2” package was used to visualize the correlation between *FAM83D* and the Polo Like Kinase 1 (*PLK1*) gene expression in differentdatasets.

### Cell culture, transfection, and reagents

MCF-7 cells were purchased from the American Type Culture Collection (ATCC; Manassas, VA, United States). We used 10% fetal bovine serum and 1% penicillin/streptomycin (Solarbio, Beijing, China) in RPMI 1640 medium (Gibco, Gland Island, NY, United States) to culture the cells. The cells were kept at 37°C in an incubator with 5% CO_2_. Jierui Biological Engineering Co., Ltd. (Shanghai, China) built the *FAM83D* knockdown vector, mixed it with the pPACKH1 packaging plasmid, and transfected it into cells. In accordance with the packaging plan described by SBI, virus particles were collected 3 days after the solution for letinous edodes concentrated virus precipitation was prepared. Infection of the cells was performed using TUNDUX viral transducers, and puromycin screening was performed to identify cells that were infected. The target sequences used were as follows: sh1-*FAM83D*: GAT​CTG​AAA​GTT​CAT​CCT​GAA and sh2-*FAM83D*: CCT​GAC​TTT​GTC​ACC​TTT​GTT.

### Quantitative reverse transcriptase-polymerase chain reaction

Using TRIzol Reagent (Invitrogen, Carlsbad, CA, United States), total RNA was extracted from the MCF-7 cells. Using PrimeScript RT Master Mix (Takara Biotechnology, Dalian, China), total RNA was reverse transcribed into cDNA, which was then used for RT- qPCR using SYBR qPCR Master Mix (Vazyme, Nanjing, China). To quantify *FAM83D* gene expression, the 2^−ΔΔCT^ was calculated for every sample and normalized to glyceraldehyde 3-phosphate dehydrogenase (GAPDH). For PCR, the *FAM83D* primers were as follows: forward: 5′-AGA​GCG​GCA​ATT​CCA​CTT​CG-3′, reverse: 5′- TGC​CAG​AAT​GAA​GGC​CAA​GG-3′. GAPDH: forward: 5′-AGA​TCC​CTC​CAA​AAT​CAA​GTG​G -3′, reverse: 5′-GGC​AGA​GAT​GAT​GAC​CCT​TTT-3′.

### Cell counting Kit-8 assay

The CCK-8 (Abcam, Shanghai, China) assay was used to measure the proliferation rate of MCF-7 cells following the manufacturer’s instructions. Further, 96-well plates were seeded with 2×10^3^ cells per well. All wells were incubated for 2 h at 37°C, followed by the measurement of absorbance at 450 nm in a microplate reader at the desired time point.

### Transwell and flow cytometry assay

After collecting cultured sh1-*FAM83D*, sh2-*FAM83D*, and negative control (NC) group cells with serum-free medium, they were counted. 1×10^4^ cells were evenly inoculated into the upper layer of each compartment and a culture medium containing 10% fetal bovine serum (500 μl) was added to the lower layer of the compartment to induce cell migration to the other side. After incubation for 24 h at 37°C in a 5% CO2 incubator, the cells were fixed in 4% methanol for 30 min, stained with 0.1% crystalline violet for 20 min, and rinsed off with PBS. Finally, five representative images were randomly captured under an inverted microscope. For the detection of cell invasion, Matrigel (BD, Bioscience, Pharmingen) was spread in advance on the upper layer of the chambers and placed in the incubator for 2–4 h. The rest of the procedure was carried out following the migration assay steps above.

For cell cycle assay, MCF-7 cells were collected and fixed in 80% pre-cooled ethanol for about 18 h at 4°C in a refrigerator. The following day, cells were resuspended with propidium staining solution and incubated at 37°C for 30 min protected from light. Ensure that the cycling assay is completed by flow cytometry within 1 h and the results are analyzed for data using ModFit software.

### Animal experiment

Female BALB/c mice that were 4-weeks old, naked, and reared in a sterile environment were used for *FAM83D* silencing experiments (Vitalriver, Beijing, China). The constructs sh-*FAM83D* and NC group were transfected into 3×10^6^ MCF-7 cells and then injected into the nude mice through the tail vein (n = 5 for each treatment group). Every 3 days, the tumor sizes were measured. The mice were euthanized after 24 days, and their tumor weight were measured. This animal experiment was reviewed and approved by the University of Science and Technology of China Animal Experimentation Ethics Committee.

## Results

### Pan-cancer FAM83D expression

**FIGURE 1:**
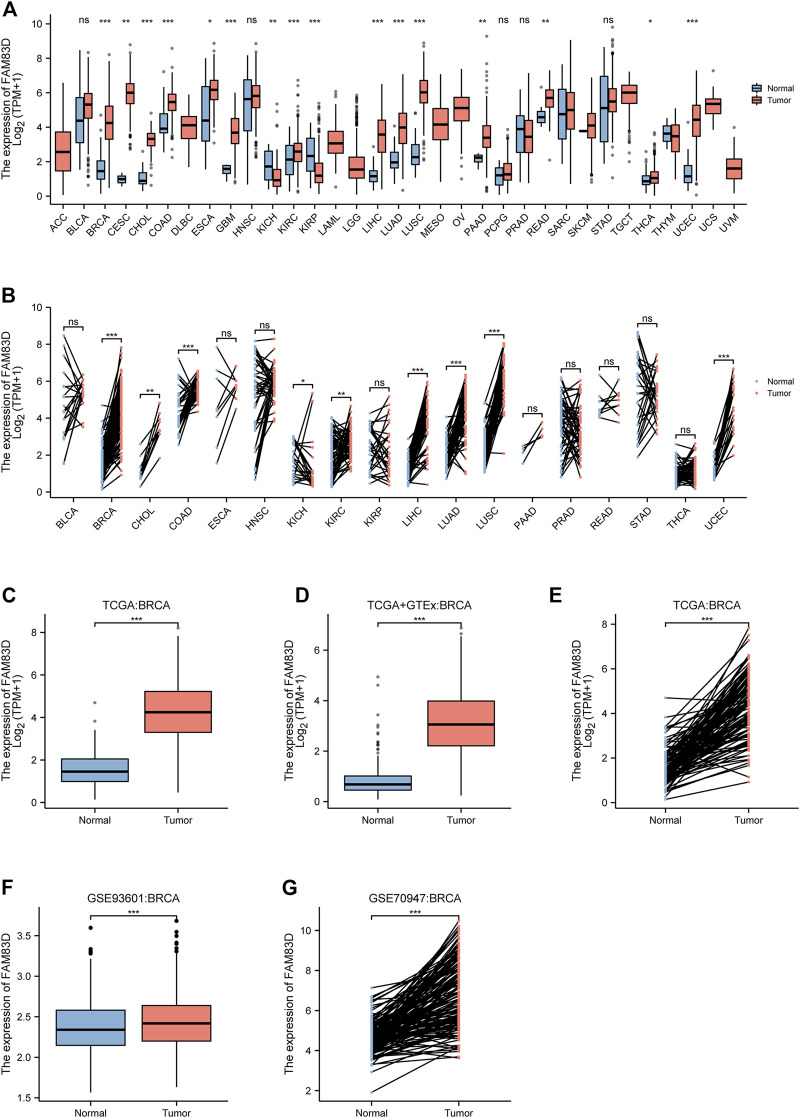
The expression level of FAM83D in human cancer **(A)** Pan-cancer analysis in TCGA cohort **(B)** Pan-cancer pair analysis in TCGA cohort **(C)** Expression in TCGA breast cancer tumour tissues and the normal **(D)** Expression in TCGA and GTEx of breast cancer **(E)** Expression in TCGA breast cancer paired samples **(F)** Expression of the GSE93601 dataset in BRCA **(G)** Expression of the GSE70947 dataset in BRCA (*p < 0.05, **p < 0.01, ***p < 0.001).

The expression of *FAM83D* was higher in cholangiocarcinoma (CHOL), colon adenocarcinoma (COAD), lung adenocarcinoma (LUAD), lung squamous cell carcinoma (LUSC), glioblastoma multiforme (GBM), kidney renal clear cell carcinoma (KIRC), rectum adenocarcinoma (READ), and uterine corpus endometrial carcinoma (UCEC) samples compared to that in normal tissue samples (*p* < 0.001; Figure 1A). Paired data also revealed that the expression level of *FAM83D* in tumor tissues exceeded that in normal tissues (Figure 1B). In addition, by comparing *FAM83D* expression data from TCGA datasets, combining the normal tissue data of GTEx with TCGA data, and reviewing paired data (Figures 1C,D), we discovered that the expression level of *FAM83D* in BRCA tissues was markedly higher than in normal tissues (*p* < 0.001). To further verify the dissimilarity in *FAM83D* expression between BRCA and normal tissues, 1,110 samples, including 602 tumor and 508 tumor-adjacent normal tissue samples, from the GSE93601 dataset in the GEO database were integrated and analyzed. These results also confirmed the conclusion that FAM83D was highly expressed in tumor tissues than in paired adjacent tissues (Figure 1F). Similarly, validating the expression data of 148 paired specimens in the external dataset GSE70947 found consistent results with the TCGA analysis (Figure 1G).

### Pan-cancer diagnostic value of FAM83D

**FIGURE 2:**
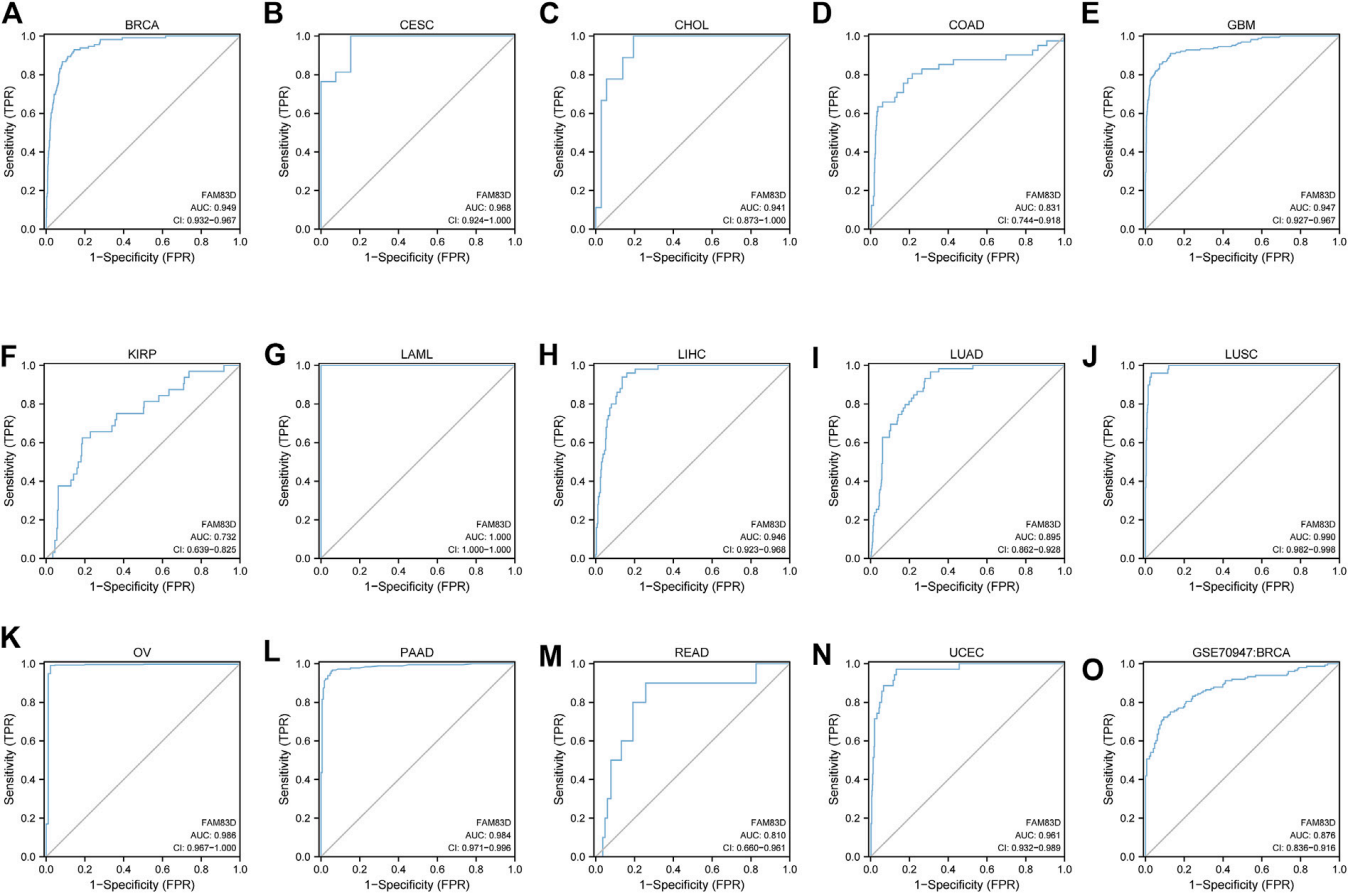
ROC curve of pan-cancer **(A–N)** ROC curve of BRCA, CESC, CHOL, COAD, GBM, KIRP, LAML, LIHC, LUAD, LUSC, OV, PAAD, READ, UCEC **(O)** ROC curve of the GSE70947 dataset in BRCA.

We next constructed a receiver operating characteristic (ROC) curve to evaluate the diagnostic value of *FAM83D* expression in the TCGA cohort data. Ourr analysis showed (Figures 2A–N) that *FAM83D* expression may act as a diagnostic marker in BRCA (area under the curve, AUC = 0.949; 95% confidence interval, CI: 0.932–0.967), cervical cancer (CESC; AUC = 0.968, 95% CI: 0.924–1.000), CHOL (AUC = 0.941, 95% CI: 0.873–1.000), COAD (AUC = 0.831, 95% CI: 0.744–0.918), GBM (AUC = 0.947, 95% CI: 0.927–0.967), kidney renal papillary cell carcinoma (KIRP; AUC = 0.732, 95% CI: 0.639–0.825), acute myeloid leukemia (LAML; AUC = 1.000, 95% CI: 1.000–1.000), liver hepatocellular carcinoma (LIHC; AUC = 0.946, 95% CI: 0.923–0.968), LUAD (AUC = 0.895, 95% CI: 0.862–0.928), LUSC (AUC = 0.990, 95% CI: 0.982–0.998), ovarian serous cystadenocarcinoma (AUC = 0.986, 95% CI: 0.967–1.000), pancreatic adenocarcinoma (PAAD; AUC = 0.984, 95% CI: 0.971–0.996), READ (AUC = 0.810, 95% CI: 0.660–0.961), UCEC (AUC = 0.961, 95% CI: 0.932–0.989). Furthermore, the ROC curve with an area of 0.876 under the subject working characteristic curve (95% CI: 0.836–0.916) indicated that *FAM83D* expression had a high degree of accuracy in diagnosing BRCA in the GSE70947 dataset (Figure 2O).

### High FAM83D expression in patients predicts poor prognosis

**FIGURE 3:**
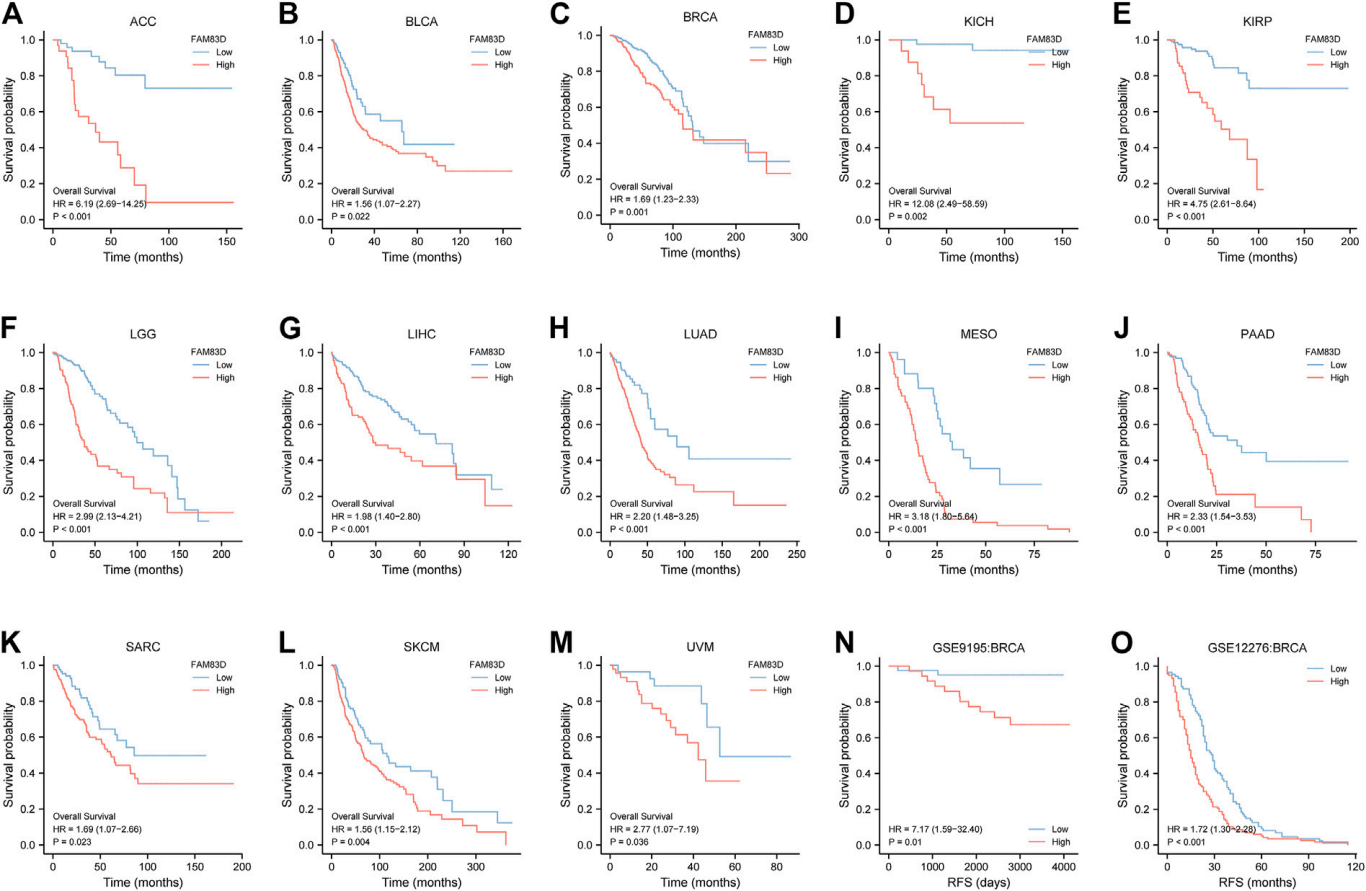
Kaplan–Meier survival curves comparing the high and low expression of FAM83D in different types of cancer in Kaplan–Meier Plotter. **(A–M)** Kaplan–Meier analysis of the association between FAM83D expression and OS in the TCGA cohort. **(N,O)** RFS analysis of RFS in GSE9195 and GSE12276 in BRCA.

As shown in Figures 3A–L, the overall survival (OS) of patients with low expression of *FAM83D* was better than for patients with high expression of *FAM83D* in adrenocortical carcinoma (ACC; *p* < 0.001), bladder urothelial carcinoma (BLCA; *p* = 0.022), BRCA (*p* = 0.001), kidney chromophobe (KICH; *p* = 0.002), KIRP (*p* < 0.001), brain lower grade glioma (LGG; *p* < 0.001), LIHC (*p* < 0.001), LUAD (*p* < 0.001), mesothelioma (MESO; *p* < 0.001), PAAD (*p* < 0.001), sarcoma (SARC; *p* = 0.023), skin cutaneous melanoma (SKCM; *p* = 0.004), and uveal melanoma (UVM; *p* = 0.036). To further validate this result, we sorted out the chip expression and recurrence-free survival (RFS) data from the GSE9195 dataset (*p* = 0.01; Figure 3N) in the GEO database. Analysis of the RFS of GSE12276 showed that patients in the high-expression *FAM83D* group had a worse prognosis than those in the low-expression *FAM83D* group (Figure 3O) (*p* < 0.001).

The Cox regression module of the R “survival” package was used to analyze BRCA data from the TCGA dataset with univariate and multivariate statistics. As shown in Table 1, univariate meaningful parameters were included in the multivariate analysis. Multivariate analysis showed that *FAM83D* expression (hazard ratio, HR = 1.641 (95% CI: 1.147–2.348)) was independently correlated with OS. Moreover, the N stage, patient age, and pathologic stage were also independent predictors of BRCA prognosis.

**TABLE 1:** Univariate and multivariate analysis of the association of FAM83D with OS in BRCA (TCGA).

Characteristics	Total(N)	Univariate analysis		Multivariate analysis	
		Hazard ratio (95% CI)	*p* value	Hazard ratio (95% CI)	*p* value
T stage	1,079				
T1&T2	905	Reference			
T3&T4	174	1.608 (1.110–2.329)	**0.012**	0.923 (0.549–1.549)	0.760
N stage	1,063				
N0	514	Reference			
N1&N2&N3	549	2.239 (1.567–3.199)	**<0.001**	1.746 (1.120–2.721)	**0.014**
M stage	922				
M0	902	Reference			
M1	20	4.254 (2.468–7.334)	**<0.001**	2.023 (1.001–4.087)	0.050
Age	1,082				
<=60	601	Reference			
>60	481	2.020 (1.465–2.784)	**<0.001**	2.279 (1.574–3.298)	**<0.001**
PR status	1,029				
Negative	342	Reference			
Positive	687	0.732 (0.523–1.024)	0.068		
ER status	1,032				
Negative	240	Reference			
Positive	792	0.712 (0.495–1.023)	0.066		
HER2 status	715				
Negative	558	Reference			
Positive	157	1.593 (0.973–2.609)	0.064		
Pathologic stage	1,059				
I& II	799	Reference			
III& IV	260	2.391 (1.703–3.355)	**<0.001**	1.819 (1.057–3.132)	**0.031**
Histological type	977				
IDC	772	Reference			
ILC	205	0.827 (0.526–1.299)	0.410		
FAM83D	1,082				
Low	540	Reference			
High	542	1.405 (1.019–1.938)	**0.038**	1.641 (1.147–2.348)	**0.007**

The bold values means: *P*-values are bolded when less than 0.05.

### FAM83D is related to immune infiltration in pan-cancer

**FIGURE 4:**
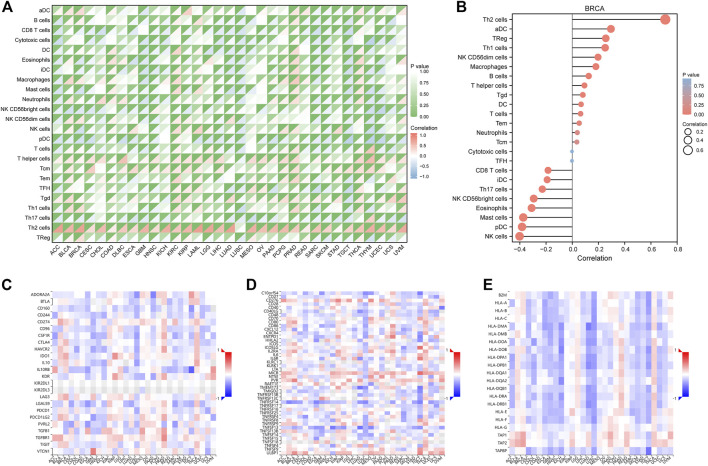
Immune-related analysis. **(A)** TCGA immune cell infiltration analysis in pan-cancer **(B)** immune cell infiltration analysis of BRCA **(C–E)** Correlation analysis between FAM83D and Immunoinhibitor, Immunostimulator and MHC molecule in the TISIDB database.

The ssGSEA algorithm was used to perform immune infiltration analysis on samples from the TCGA dataset. Spearman correlation analysis showed the correlation between *FAM83D* expression in the tumor environment and immune cells (Figure 4A). Th2 and T helper cells were positively correlated with *FAM83D* expression in most cancer types. In contrast, in BRCA, KIRC, and prostate adenocarcinoma (PRAD), the degree of immune cell infiltration was correlated with the expression level of *FAM83D*. In the ssGSEA results for BRCA (Figure 4B), the expression level of *FAM83D* was positively correlated with Th2 cells and aDC cells but negatively correlated with Natural Killer (NK) cells.

We further analyzed the relationship between *FAM83D* expression and Immunoinhibitor, Immunostimulator, and MHC molecules in the TISIDB database (Figures 4C–E). Our results revealed that *FAM83D* expression is negatively associated with immune-related molecules in LUSC and KICH and positively associated with these molecules in BRCA, PRAD, and thyroid carcinoma.

### Single-cell sequencing analysis

**FIGURE 5:**
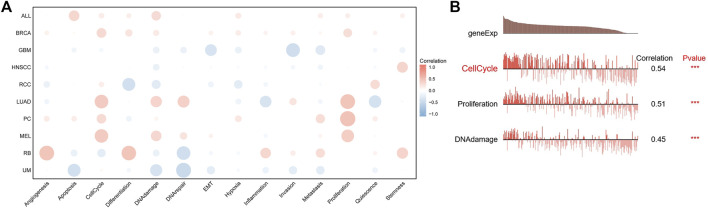
The correlation between FAM83D expression and 14 cancer functional states using single-cell sequence data from the CancerSEA database (**(A)** The correlation between CENPL expression and 14 cancer functional states in pan-cancer **(B)** The expression of CENPL is positively correlated with the DNA repai, cell cycle, DNA damage, proliferation and Hypoxia of BRCA (***p < 0.001).

Next, we analyzed the correlation between *FAM83D* expression and 14 cancer functional states using single-cell sequencing data from CancerSEA. *FAM83D* expression was positively associated with the cell cycle in most tumor types (Figure 5A), whereas it was negatively associated with hypoxia (Figure 5A). As for BRCA, there was a significant positive correlation between *FAM83D* expression and the cell cycle (cor = 0.54, *p* < 0.001), DNA damage (cor = 0.45, *p* < 0.001) and proliferation (cor = 0.51, *p* ≤ 0.001) (Figure 5B).

### Functional analysis of FAM83D in BRCA

**FIGURE 6:**
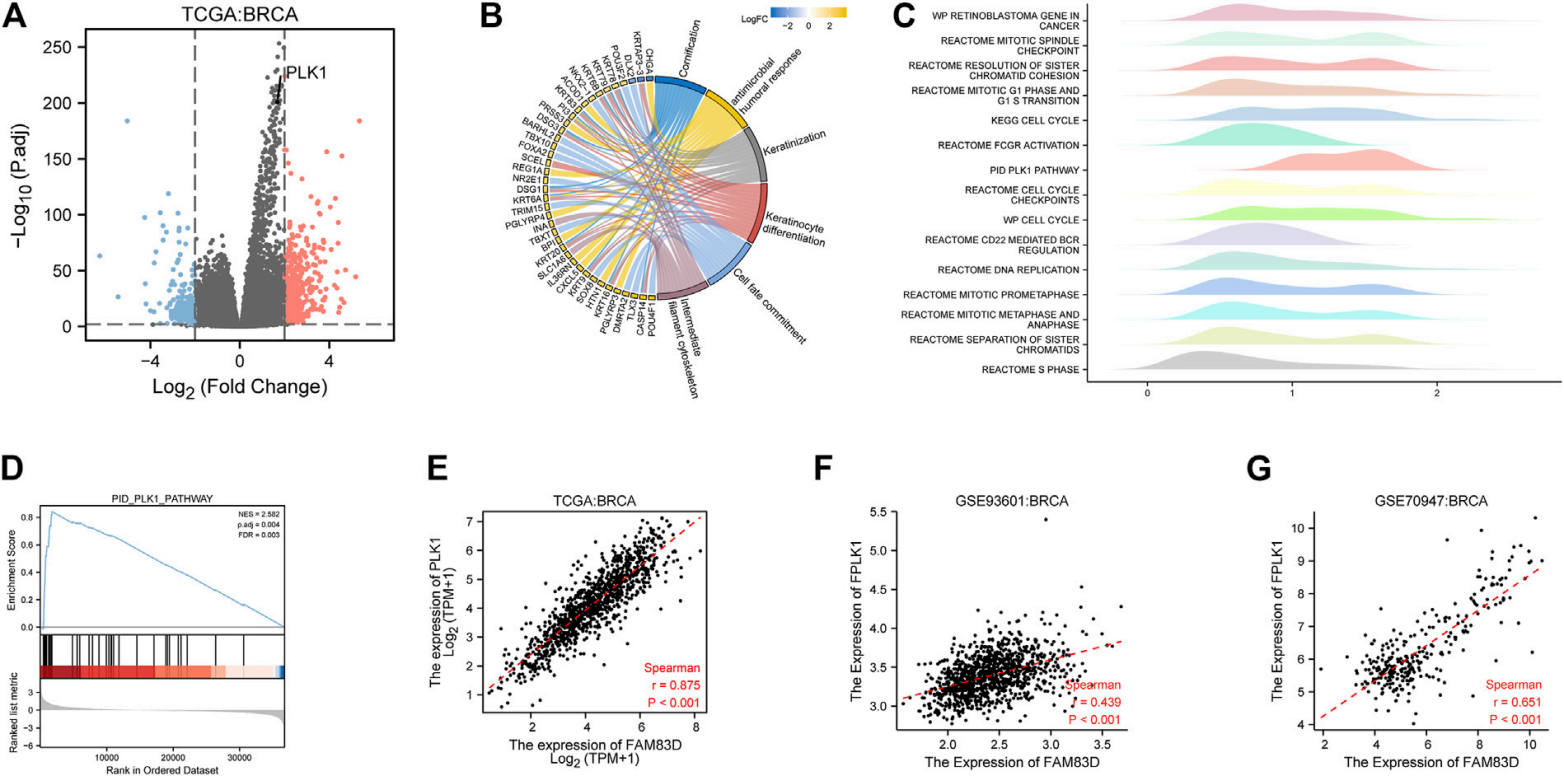
Pathway enrichment and correlation analysis in BRCA. **(A)** Differential genes in the TCGA cohort. **(B)** KEGG and GO analyses of the top ranked pathways. **(C)** Pathway enrichment by GSEA. **(D)** PLK1 pathway of GSEA. **(E–G)** Correlation between the FAM83D and PLK1 of TCGA, GSE93601 and GSE70947.

We next used the median expression level of *FAM83D* in BRCA samples as a grouping cutoff value and displayed the differentially expressed genes (LogFC = 2, *p* < 0.01) in a volcano plot in Figure 6A. We found that the *PLK1* gene was significantly elevated in the high expression group of *FM83D*. Subsequently, GO and KEGG enrichment analyses showed that the differentially expressed genes were primarily enriched in the cornification, antimicrobial humoral response, keratinization, keratinocyte differentiation, cell fate commitment, and intermediate filament cytoskeleton pathways (Figure 6B). GSEA pathway enrichment analysis further indicated that the cell cycle, S phase, PLK1, and DNA replication pathways are related to *FAM83D* expression in BRCA (Figures 6C,D). Molecular correlation analysis of BRCA data from the TCGA, GSE93601, and GSE70947 datasets showed that the expression level of PLK1 was strongly correlated with *FAM83D* expression (Figures 6E–G). As shown in Supplementary Figure S1, we further verify the relationship between *FAM83D* and PLK1 in other tumors by biogenic analysis. Consistent with BRCA results, a total of 25 cancers, *FAM83D* expression levels strongly correlated with PLK1.

### Verification of FAM83D results using RT‒qPCR and CCK-8 assay

**FIGURE 7:**
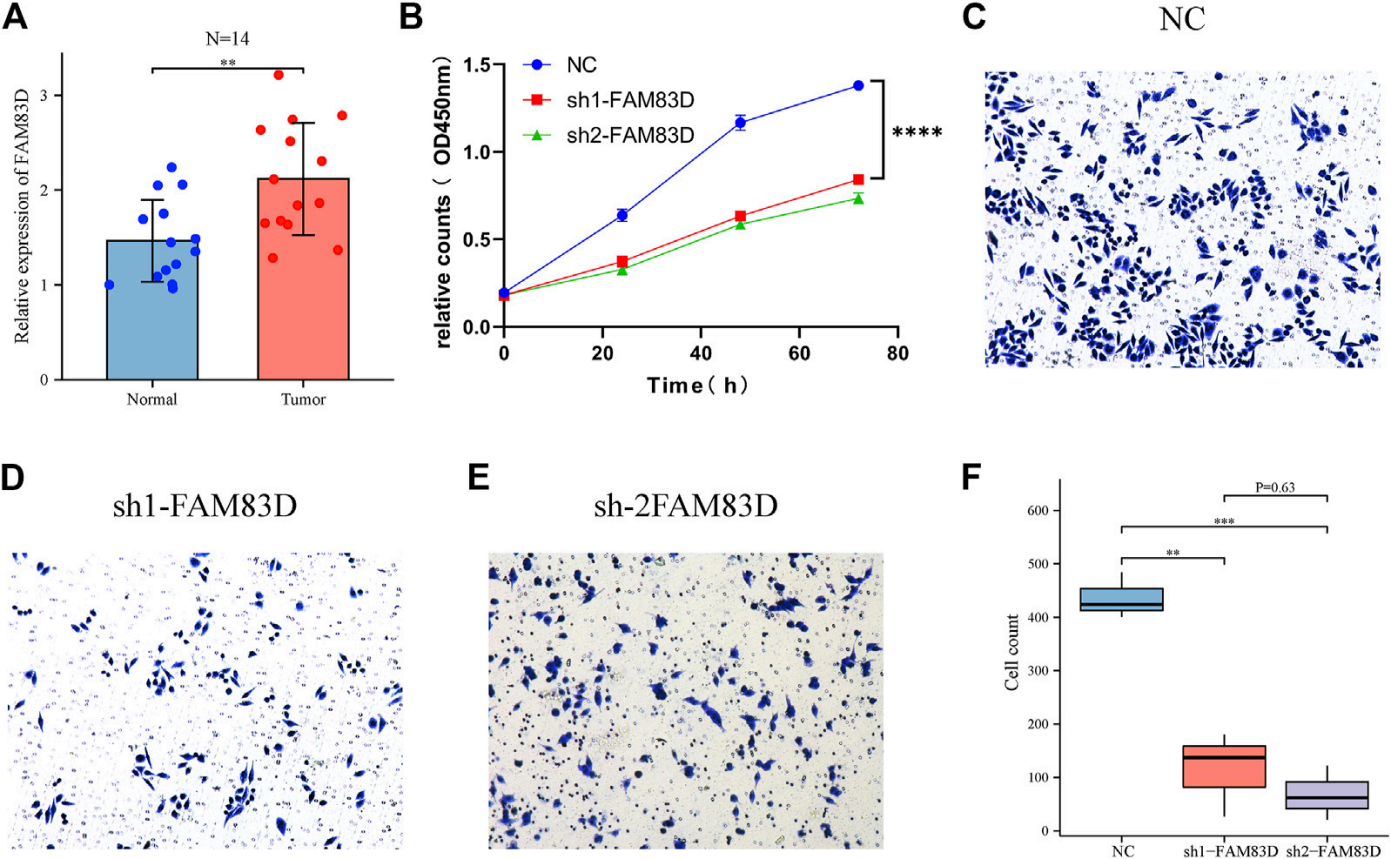
FAM83D was verified by RT‒qPCR, CCK-8 and transwell assay in MCF-7 cells. **(A)** RT‒qPCR analysis of tumor FAM83D expression in comparison to normal cells. **(B)** CCK-8 assay demonstrated that FAM83D knockdown prevented the growth of MCF-7 cells. **(C–F)** Transwell assay showed that knockdown of FAM83D significantly inhibited the invasion and migration ability (**p < 0.01, ***p < 0.001, ****p < 0.0001).

RT‒qPCR results showed that *FAM83D* mRNA expression level in 14 pairs of BRCA tissue samples was higher than in adjacent normal tissue samples (*p* < 0.01, Figure 7A). This confirmed the results of our bioinformatics analysis of the TCGA, GSE93601, and GSE70947 datasets. Additionally, the CCK-8 assay revealed a significant difference between the knockdown *FAM83D* and NC groups in terms of cell proliferation (Figure 7B).

### Verification of FAM83D results using transwell and the flow cytometric assay

**FIGURE 8:**
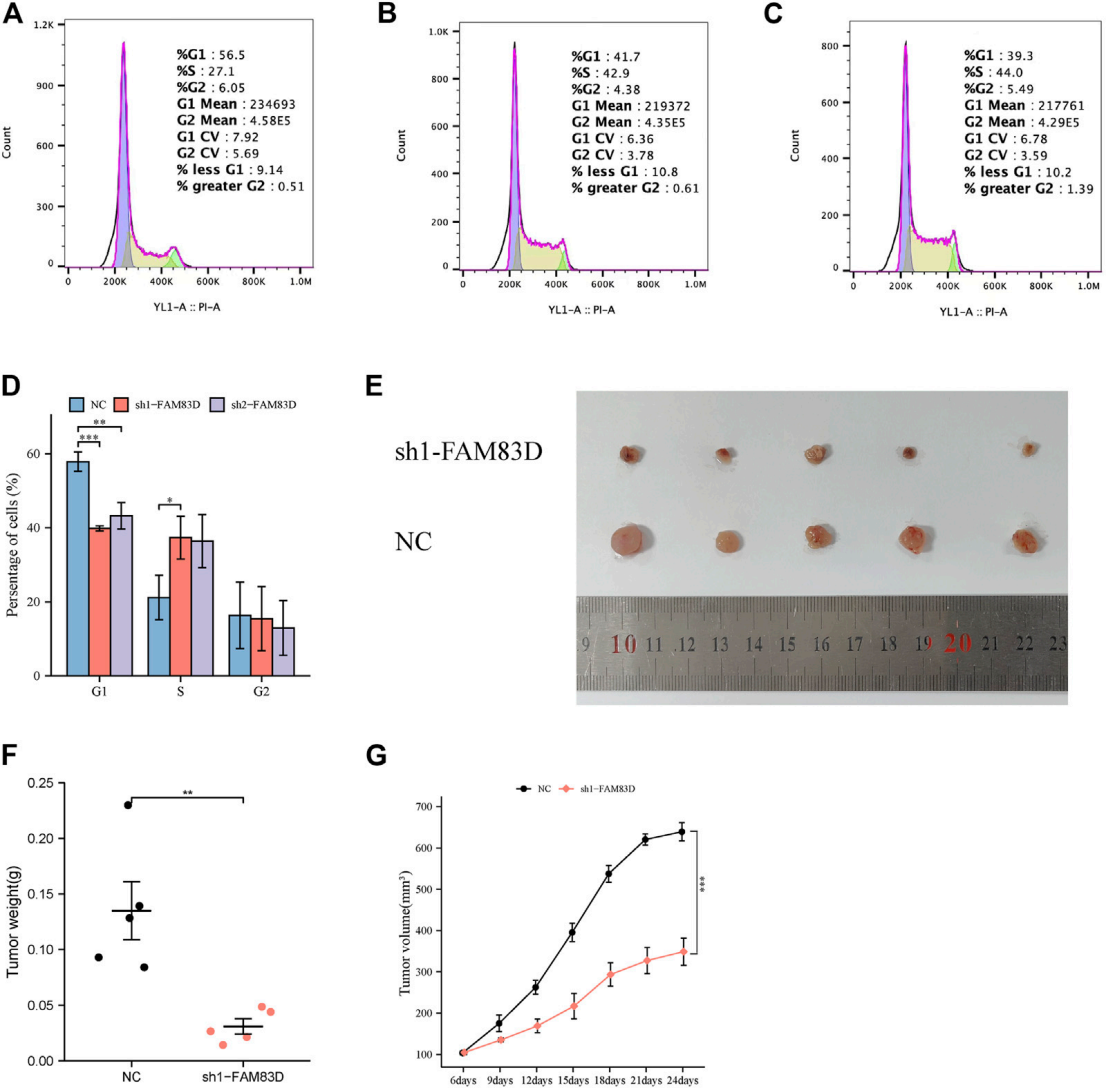
Flow cytometry assay and animal experiment. **(A–D)** Flow cytometric assays revealed that knockdown of FAM83D blocked cell cycle. **(E–G)** Knockdown of FAM83D inhibits tumor growth *in vivo* (*p < 0.05, **p < 0.01, ***p < 0.001).

Transwell assay showed that knockdown of *FAM83D* significantly inhibited the invasion and migration ability of BRCA cells compared to control (Figures 7C–F). Moreover, Fflow cytometric assays revealed that knockdown of *FAM83D* can alter the cell cycle by arresting the G1 phase (Figures 8A–D). This is consistent with previous pathway enrichment results, suggesting that *FAM83D* may play a role in affecting the proliferation of MCF-7 cells by regulating the cell cycle.

### Knockdown ofFAM83D inhibits tumor growth *in vivo*

To further validate the function of *FAM83D*, we divided nude mice into sh1-*FAM83D* and NC groups. Tumor volume was measured every 3 days starting at approximately 1 week after MCF-7 cell injection. Tumor volume in the sh1-*FAM83D* group was much lower than that of the NC group, as shown in Figure 8E. Mice were then euthanized and their tumors were 24 days after cell injection. These tumorigenicity experiments revealed that silencing *FAM83D* had a lower tumorigenic potential than was shown for the control mice. The size, weight, and volume of the tumors in the treatment group were considerably lower than those in the NC group (Figures 8F,G).

## Discussion

It has previously been reported that *FAM83D* is involved in various complex biological processes, including cell proliferation, apoptosis, and invasion (Cooper et al., 2020; Yin et al., 2020; Yu et al., 2020). In addition, emerging studies have suggested a functional link between *FAM83D* and tumor development (Meng et al., 2021; Zhao et al., 2022). However, there is no unified conclusion on how *FAM83D* plays a role in different tumor types or the underlying mechanisms. To our knowledge, there is currently no pan-cancer analysis of *FAM83D*. Therefore, we comprehensively analyzed the role of *FAM83D* expression in various tumor types, with a special focus on BRCA, from the perspectives of gene expression, prognosis, immune infiltration, and related pathways using multiple cohort datasets.

To analyze *FAM83D* expression among different cancer types, we examined pan-cancer differential *FAM83D* expression between cancer tissue samples and their matched normal tissue samples from the TCGA dataset. First, we investigated the expression level of *FAM83D*. We found that in most cancer types, such as COAD, LUAD, LUSC, GBM, and BRCA, *FAM83D* expression in tumor tissue was significantly higher than in normal tissue. Subsequently, based on survival data from the TCGA, we found a highly consistent trend between poor patient prognosis and high *FAM83D* expression in multiple tumor types, including BRCA. These findings were further verified in the independent dataset of BRCA in the GEO database.

Next, we focused on exploring the role of *FAM83D* in BRCA. GSEA enrichment analysis of BRCA data in TCGA showed that genes from proliferation and cell cycle-related pathways were more likely to be enriched in the high *FAM83D* expression BRCA cohort. In addition, single-cell analysis demonstrated that *FAM83D* expression is strongly associated with cell proliferation and cell cycle in BRCA. CCK-8 assay also demonstrated this functionality. It is worth mentioning that the PLK1 pathway is closely related to the expression level of *FAM83D*. In addition, in the molecular correlation analysis of TCGA, GSE93601, and GSE70947, BRCA samples showed a strong positive correlation between the expression of *PLK1* and *FAM83*D. This suggests that *FAM83D* may play a role in promoting tumorigenesis by affecting the PLK1 pathway. The PLK1 pathway has previously been shown in a large number of studies to be closely related to a variety of cancers, including BRCA (Mao et al., 2021; Shi et al., 2016), and plays a role in multiple biological processes such as BRCA cell proliferation, drug resistance, and metastasis (Chiappa et al., 2022; Qian et al., 2011; Wang et al., 2021).

In the treatment of patients with breast cancer, immunotherapy plays a critical role, and the continuous emergence of new biomarkers adds to the early and successful treatment of BRCA. The expression of *FAM83D* was firmly related to immune cells such as Th2 cells through our pathway enrichment and immune infiltration analyses. There is evidence that Th2 cells can promote tumorigenesis and progression, thus leading to poor tumor immunotherapy efficacy. This may one reason for the poor survival outcomes seen in patients with high expression of *FAM83D* (Espinoza et al., 2016). In contrast, there was a negative correlation between the expression level of *FAM8D* and NK cells. As powerful innate immune cells with multiple mechanisms to kill cancer cells, NK cells play a crucial role in the immune response to cancer. Therefore, further studies are needed to reveal the complex relationship between *FAM83D* and NK cells in BRCA (Lee et al., 2021).

Despite our comprehensive and detailed analysis of *FAM83D*, this study mainly focuses on the pan-cancer expression of *FAM83D* and its prognostic significance. There is a need for further research on *FAM83D* protein levels and the specific molecular mechanisms of *FAM83D* function in BRCA.

## Conclusion

In sum, our study demonstrates the important pan-cancer role of *FAM83D* gene expression, especially in BRCA. Thus, it may serve as a key prospective prognostic biomarker of BRCA.

## Data Availability

The original contributions presented in the study are included in the article/Supplementary Material, further inquiries can be directed to the corresponding authors.
