# Supplementary figures and images for: Pan-cancer and single-cell analysis reveals FAM83D expression as a cancer prognostic biomarker
Source: Front Genet. 2022 Dec 9;13:1009325. doi: 10.3389/fgene.2022.1009325 (PMC9780495; doi:10.3389/fgene.2022.1009325)

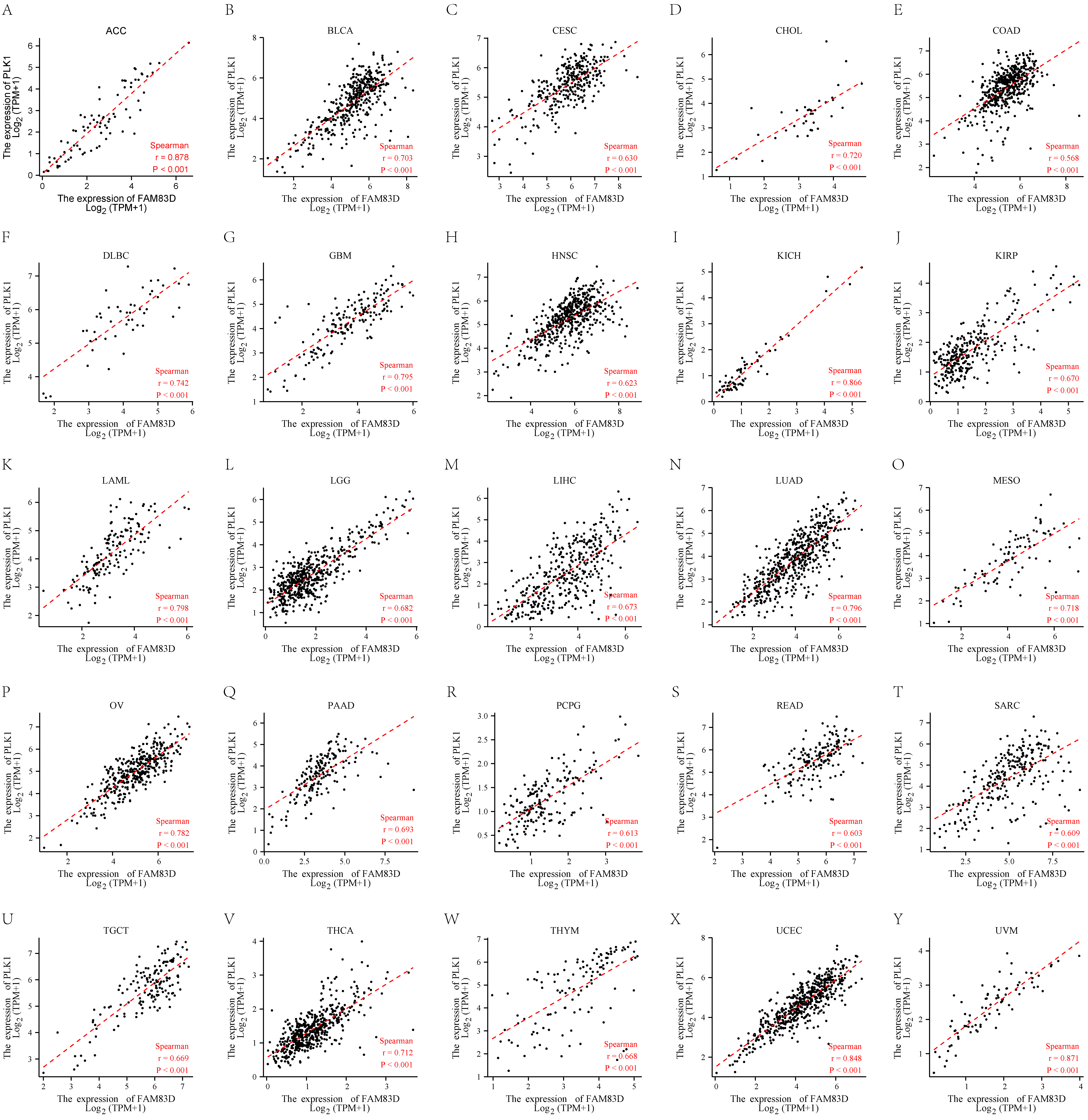

Supplement: Supplementary file 1 [file Image1.TIF]
